# Assessing the effect of health status on multidimensional poverty among older adults: the Chinese longitudinal healthy longevity survey

**DOI:** 10.3389/fpubh.2023.1150344

**Published:** 2023-07-05

**Authors:** Lulin Zhou, Change Zhu, Christine A. Walsh, Xinjie Zhang

**Affiliations:** ^1^Department of Management, Jiangsu University, Zhenjiang, China; ^2^Faculty of Social Work, University of Calgary, Calgary, AB, Canada

**Keywords:** multidimensional health, multidimensional poverty, older adults, medical expenditure, China, CLHLS

## Abstract

**Background:**

This study aimed to explore the association between health status (physical, mental, and self-rated health) and multidimensional poverty (subjective and objective poverty) in older adults.

**Method:**

A panel binary logit regression approach was applied to four waves of CLHLS data (2008, 2011, 2014, and 2018). In total,1,445 individuals were included after data cleaning.

**Results:**

The mean values and proportion of physical, mental, and self-rated health were 5.73 (87.42%), 0.93 (93.06%), and 3.46 (86.7%), respectively, and mean values and proportion of subjective and objective poverty were 0.19 (18.51%) and 0.21(21.4%). In addition, physical, mental, and self-rated health were all found to be associated with subjective poverty among older adults (*r* = −0.181, *r* = −0.630, *r* = −0.321, *p* < 0.05), that is, the better the physical, mental, and self-rated health, the lower the probability of subjective poverty. A comparable connection between self-rated health and objective poverty also exists (*r* = −0.157, *p* < 0.05). Furthermore, medical expenditure played a mediation role in the association between the health status and poverty of older adults.

**Conclusion:**

In order to effectively alleviate the poverty of older adults, strategies should be taken to improve the health level of older adults, especially the physical and mental health of high-aged older adults, and the self-rated health of middle-aged older adults. Furthermore, social security and pensions should be further developed to adequately reimburse medical expenditures.

## 1. Research background

Older adults are a vulnerable group in society in relation to poverty (1). Based on the poverty cycle theory, poverty is more likely to occur in older adults (2). As the world’s population ages, the issue of poverty among older adults has become increasingly prominent. China has the largest aging population in the world, as noted in previous studies (3, 4). It is estimated that China will have the largest older population in the world by 2050, with a projected number of 488 million older adults, comprising 35.5% of the total population.^1^ Hence, it is imperative to accord significant attention to the issue of poverty among older adults in China.

Poverty not only imposes a significant burden on society, but also leads to a wide range of negative outcomes, including reduced access to healthcare (5), inadequate nutrition, and limited opportunities for social engagement. Under the circumstances, the high poverty rate of older adults is serious and needs to be addressed urgently (6, 7).

The multifaceted nature of poverty is widely acknowledged, as it can be defined and measured using a variety of dimensions (8). To our knowledge, economic poverty is a common experience for the older population (9). The comprehensive rollout of basic medical insurance plans in China has effectively addressed objective poverty concerns, however, due to the persistence of wealth inequality, the perception of poverty remains a primary issue. As such, it is necessary to conduct a comprehensive analysis of both objective and subjective poverty, that is, multidimensional poverty, among older adults (8).

Available studies have shown that older adults’ poverty is related to individual-level, community-level, and policy-level factors such as age (10), gender (11), education (12, 13), marital status (14, 15), occupation (16), and place of residence (17). Furthermore, neighborhood service and community service are factors affecting poverty (18). Also, deficiency of government support and social security (19) are found to be closely related to poverty.

In recent years, there are an increasing number of researchers that are focusing on the impact of poverty on the health of older adults. However, few studies are concerned about the effect of health on poverty among older adults. The theory of poverty raised by Sen proposed that capability deprivation is the main cause of poverty (20). It was suggested that the ability of older adults typically decreases with aging due to the degradation of physical and cognitive function (21), thus resulting in a deficiency in income (22). Additionally, income level is normally found to be related to poverty among older adults (23). Thus, it can be supposed that improving health is one of the key initiatives to reduce poverty. Having good health is the basis and premise for individuals to participate in the labor market. It is well acknowledged that the health status of older adults is multidimensional, including not only physical health but also psychological and self-rated health. Therefore, it is necessary to conduct research that enriches our understanding of the multidimensional relationship between the health status of older adults and their poverty status.

In addition, some scholars have shown that the worse a population’s health status, the more morbidities, and the heavier the medical expenditure, thus resulting in an economic burden (24, 25). In China, poverty related to disease is prevalent, particularly among older adults (26). According to statistics, the number of households returning to poverty as a result of the disease fell from 7.269 million at the end of 2015 to 3.882 million in 2017 in China.^2^ Although the proportion of poverty caused by disease has decreased, the phenomenon of poverty caused by medical expenses remains severe.

Thus, the primary objective of this study was to examine the health status (including physical, mental, and self-rated health) and poverty (subjective and objective poverty) among older adults in China. Moreover, it aimed to investigate the impact of health status on poverty and explore the role of medical expenses in influencing this relationship.

## 2. Methods

### 2.1. Data and sample

Data were derived from the 2008, 2011, 2014, and 2018 Chinese Longitudinal Healthy Longevity Surveys (CLHLS). The survey was organized by the Research Center of Healthy Aging and Development of Peking University/National Development Institute. This survey is a comprehensive longitudinal study conducted across 23 provinces/autonomous regions in China. The regions were selected randomly for in-person household interviews. This survey mainly focuses on older adults. The respondents in this article were the older adults who participated in the research in 2008, 2011, 2014, and 2018. The survey groups were mainly older people aged 60 and older. In total, 1,445 individuals were investigated after data cleaning.

### 2.2. Variable definitions

#### 2.2.1. Poverty (dependent variable)

The poverty of older adults was the dependent variable in this research. Different from the solo measure of economic poverty studied by previous research (27), the poverty of older adults in this article included both subjective and objective poverty. Subjective poverty was reflected by “whether all your sources of living are sufficient,” with answers of 0 identified as non-subjective poverty and 1 identified as subjective poverty. Similarly, objective poverty was reflected by whether the *per capita* total income of residents reached the *per capita* annual income standard of 1,196 RMB in 2008, 2,300 RMB in 2011, 2,800 RMB in 2014, and 3,535 RMB in 2018. If the *per capita* annual income exceeded the criteria, it was determined as non-objective poverty, coded as 0; otherwise, it was identified as objective poverty, coded as 1.

#### 2.2.2. Health status (independent variable)

The health status of older adults was the independent variable in this article. According to the extant research, health is not only physical function without disease but also good mental condition (28). In addition, self-rated health is found to be valid for people’s assessments of their general health (29). Therefore, physical, mental, and self-rated health were utilized to measure the multidimensional health status of older adults in this research. Specifically, physical health refers to the unimpaired body function of the individual, which can ensure the normal progress of daily activities. The physical health of older adults is reflected by six activities of daily living (ADL) of “bathing, dressing, going to the toilet, indoor activities, and fecal control and eating” (30) and if the participants were able to perform the ADL independently, it was recorded as 1; otherwise, it was recorded as 0. The total score of these six activities is used as an indicator of physical health for older adults. Thus, the score range was 0–6. In addition, they were considered to be physically healthy if the six routine activities were completed independently. If not, they were considered to be physically unhealthy.

Furthermore, the World Health Organization (WHO) defines mental health as a condition of overall wellness in which individuals can recognize and use their own abilities, manage typical life stressors, perform effectively and efficiently, and add value to their community (31). Mental health in this study was expressed by the personality, emotional characteristics, and depression of older adults, through the following six questions: “Do you always look on the bright side of things?,” “Do you often feel fearful or anxious?,” “Do you often feel lonely and isolated?,” “Can you make your own decisions concerning your personal affairs?,” “Do you feel the older you get, the more useless you are?,” and “Are you as happy as when you were younger?” All the items were a five-level Likert scale; negative scoring items were transferred into positive ones (1 = very unhealthy, 2 = relatively unhealthy, 3 = average, 4 = relatively healthy, and 5 = very healthy). Specifically, if the total score for mental health was greater than 18 or equal to 18, it was identified as mentally healthy and coded as 1. Otherwise, if the score is less than 18, it was identified as mentally unhealthy and coded as 0.

Self-rated health is a comprehensive variable widely used in health status measurement. It is a general comprehensive evaluation made by the respondents based on their subjective cognition of their own health status (32, 33). Thus, in this article, self-rated health was reflected by older adults’ assessment of their own health status, measured by the item, “How do you rate your health at present.” Respondents’ answers ranged from “very unhealthy”(=1), “relatively unhealthy”(=2), “average”(=3), “relatively healthy”(=4), and “very healthy”(=5). In addition, if the score of self-rated health was less than 3, it was considered to be self-rated unhealthy, otherwise, it was considered to be self-rated healthy.

#### 2.2.3. Covariates

According to the existing research, individual characteristics that affect the poverty of older adults were used as control variables. Consideration was also given to the health behaviors of older adults, such as smoking and drinking alcohol. In addition, environmental level factors were also taken into account, such as community services and social security. Details are shown in Table 1.

**Table 1:** The definition of variables and descriptive statistics.

	Variable	Variable definition	Mean (Proportion)	SD
Independent variable	Physical health	Total score of ADL ability (1–6)	5.73 (87.42%)	0.92
	Mental health	Healthy = 1, Unhealthy = 0	0.93 (93.06%)	0.25
	Self-rated health	Total score of self-rated health (1–5)	3.46 (86.7%)	0.89
Dependent variable	Subjective poverty	Poverty = 1, Non poverty = 0	0.19 (18.51%)	0.39
	Objective poverty	Poverty = 1, Non poverty = 0	0.21 (21.4%)	0.41
Mediator variable	Medical fee	Ln (medical fee)	5.74	3.38
Control variables	Age	The age of older adults (61–106)	78.97	8.83
	Gender	Male = 1, Female = 0	0.51 (51.09%)	0.50
	Residence	City = 1, Rural = 0	0.08 (8.44%)	0.28
	Living arrangement	living with family = 1; living alone = 2; living in an institution = 3	1.03	0.19
	Education	Years of schooling (0–20)	2.82	3.74
	Marital status	Currently married =1, Unmarried = 0	0.66 (66.02%)	0.47
	Smoking	Smoked = 1, never smoked = 0	0.37 (37.27%)	0.48
	Alcohol drinking	Drunk = 1, never drunk = 0	0.33 (32.75%)	0.47
	Community services	The total kinds of community service (0–8)	1.31	1.78
	Social security	The total kinds of social security (0–7)	1.34	0.79

SD denotes standard deviation.

### 2.3. Method of data analysis

The dependent variable of this study was multidimensional poverty, a binary variable, so this article used panel binary logit regression analysis to create the function logit $\left(Y\right)=a+\overset{k}{\underset{i=1}{\sum }}\beta_{i}x_{i}+\varepsilon$ . Therefore, the analysis model of influencing factors of poverty of older adults is as follows:


(1) $$P\left(Y_{j}=1\right)=\frac{\exp\left(\hat{a}+\sum_{i=1}^{k}\beta_{i}x_{i}\right)}{1+\exp\left(\hat{a}+\sum_{i=1}^{k}\beta_{i}x_{i}\right)}$$

Specifically, $P\left(Y_{j}=1\right)$ is the probability of poverty of older adults; $\beta_{i}$ is the regression coefficient of each independent variable (including the covariates); $x_{i}$ is the independent variable (including the control variable), and α is the intercept term. The poverty of older adults included subjective poverty and objective poverty. Therefore, a regression model is constructed as follows:


(2) $$P_{i}\left(SP_{i},OP_{i}\right)=\delta_{1}HS_{i}+\delta_{2}IF_{i}+\varepsilon$$

Among them, $P_{i}$ represents the probability of poverty of older adults; $SP_{i}$ and $OP_{i}$ represent the probability of subjective poverty and objective poverty, respectively; $HS_{i}$ is the health status matrix that affects the poverty of older adults and $\delta_{1}$ is its regression coefficient; $IF_{i}$ is the personal characteristic matrix and $\delta_{2}$ is its regression coefficient; ε is random disturbance term.

## 3. Results

### 3.1. Descriptive statistical analysis of the sample

From Table 1, it can be seen that the average score for physical health among older adults was 5.73, the average score for mental health was 0.93, and the average score for self-rated health was 3.46. In addition, the mean score for subjective poverty was 0.19, accounting for 18.51%, and the mean score for objective poverty was 0.21, accounting for 21.4%. In addition, the average age of older adults in this study was 78.97, with slightly more males than females, with males accounting for 51%. They mostly lived in rural areas, accounting for 91.56%, and had a generally low educational level with an average of 2.82 years of education. Regarding marital status, the majority of older adults (66%) were married at present, and the percentage of smokers and drinkers was relatively low, accounting for 37 and 33%, respectively. Furthermore, around half of the older adults (50.6%) received at least one community service. Finally, the majority of the older adults enjoyed some level of social security, accounting for 91.16%.

### 3.2. Analysis of the effect of multidimensional health status on multidimensional poverty

Table 2 shows the influence of health status and covariates on subjective and objective poverty of older adults. To control for errors and endogeneity issues, this study utilized panel data and employs fixed effects to examine the impact of health status on poverty. To be specific, Models 1 and 3 mainly investigate the effect of covariates on subjective poverty and objective poverty, respectively. Models 2 and 4 investigate the effect of health status on subjective poverty and objective poverty of older adults on the basis of covariates, respectively.

**Table 2:** The effect of health status on the poverty of older adults.

	(1)*SP*	(2)*SP*	(3)*OP*	(4)*OP*
Age	−0.043^***^(0.012)	−0.059^***^ (0.013)	0.082^***^ (0.012)	0.075^***^ (0.012)
Residence	0.685^*^ (0.306)	0.759^*^ (0.312)	−0.065 (0.303)	−0.066 (0.304)
Living arrangement	−0.095 (0.234)	−0.04 (0.239)	−0.115 (0.204)	−0.109 (0.204)
Education	−0.069^*^ (0.027)	−0.058^*^ (0.028)	−0.038 (0.024)	−0.037 (0.024)
Marriage	0.371^*^ (0.178)	0.496^**^ (0.183)	0.289 (0.182)	0.327 (0.184)
Smoking	−0232 (0.197)	−0.256 (0.200)	0.049 (0.173)	0.037 (0.173)
Alcohol drinking	0.234 (0.161)	0.265 (0.164)	0.091 (0.151)	0.096 (0.152)
Community services	−0.166^***^(0.032)	−0.157^***^ (0.033)	−0.017 (0.029)	−0.012 (0.029)
Social security	−0.024(0.076)	−0.017 (0.077)	−0.185^*^ (0.074)	−0.178^*^ (0.075)
Medical fee	0.063^***^(0.015)	0.048^**^ (0.016)	−0.030^*^ (0.014)	−0.037^**^ (0.014)
Physical health		−0.181^***^ (0.051)		−0.076 (0.050)
Mental health		−0.630^***^ (0.164)		−0.302 (0.170)
Self-rated health		−0.321^***^ (0.059)		−0.157^**^ (0.056)

Standard errors in parentheses; ^*^p < 0.5, ^**^p < 0.01, ^***^p < 0.001; SP indicates subjective poverty; OP indicates objective poverty; The same below.

Table 2 shows that age had a significant negative impact on the subjective poverty of older adults, and a significant positive impact on objective poverty, that is, the older the individual, the lower the probability of subjective poverty and the higher the probability of objective poverty. The place of residence of older adults had a significant positive impact on their subjective poverty, indicating that the probability of subjective poverty among older people living in urban areas was significantly higher than that of those living in rural areas. In contrast, the place of residence had no significant negative impact on the objective poverty of older adults. Education had a significant negative relationship with the subjective poverty of older adults, that is, the higher the education level, the lower the probability of subjective poverty. The marital status of older adults was positively correlated with subjective poverty, indicating that the subjective poverty rate of older people with spouses was significantly higher than that of those without spouses, which is contrary to previous research (34). Smoking and drinking had no significant impact on subjective and objective poverty. In addition, community services had a significant negative impact on subjective poverty, suggesting that older people who enjoy community services had a lower prevalence of subjective poverty. Social security had no significant impact on subjective poverty but had a significant negative impact on objective poverty, that is, social security significantly reduced the probability of objective poverty among older adults. Furthermore, medical expenses had a significant positive impact on subjective poverty, but a significant negative impact on objective poverty, indicating that the higher the medical expenses, the higher the probability of subjective poverty among older adults, but the lower the probability of objective poverty. Finally, the levels of physical and mental health both had a significant negative impact on subjective poverty and no significant impact on objective poverty. Self-rated health had a significant negative impact on both subjective and objective poverty, indicating that the higher the self-rated health level, the lower the probability of subjective and objective poverty among older adults.

### 3.3. Analysis of the differences in health and poverty among older adults of different age groups

In order to investigate whether age affects the health and poverty status of older adults, individuals were categorized into a low-aged group, a middle-aged group, and a high-aged group according to WHO standards. To be specific, the low-aged group included older individuals aged 60–74, the middle-aged group included those aged 75–89, and the high-aged group included individuals who were 90 years or older (35).

Table 3 presents the analysis of the health status and poverty of older adults in different age groups. The prevalence of physical and mental health problems in older adults exhibits a gradual upward trend, with a successive rise observed in the low-aged, middle-aged, and high-aged groups. Notably, the observed differences have been confirmed to be statistically significant (*p* < 0.05). The self-rated unhealthy proportion across different age groups was not found to be statistically significant (*p* > 0.05). In addition, there was a statistically significant difference in the subjective poverty proportion observed among different age groups. Specifically, a higher proportion of low-aged older adults reported subjective poverty compared to middle-aged older adults, and a higher proportion of middle-aged older adults reported subjective poverty compared to high-aged older adults (*p* < 0.05). Also, a statistically significant difference was observed in the objective poverty proportion among different age groups. Specifically, the high-aged older adults had a higher proportion of objective poverty compared to the middle-aged older adults, and the middle-aged older adults had a higher proportion of objective poverty compared to the low-aged older adults (*p* < 0.05). Thus, the poverty status of older adults varied significantly across different age groups.

**Table 3:** Health and poverty analysis of different age groups.

		High-aged (> = 90)	Middle-aged (75–89)	Low-aged (60–74)	Chi-square	*p* value
Health status	Physical unhealthy	259 (35.14%)	374 (12.19%)	94 (4.76%)	451.4787	0.000
	Mental unhealthy	73 (9.91%)	229 (7.46%)	99 (5.01%)	22.704	0.000
	Self-rated unhealthy	81 (10.99%)	427 (13.92%)	261 (13.22%)	4.4357	0.109
Poverty status	Subjective poverty	124 (16.82%)	546 (17.80%)	400 (20.25%)	6.4005	0.041
	Objective poverty	173 (23.47%)	686 (22.36%)	378 (19.14%)	9.5651	0.008

### 3.4. Robust test

In order to enhance the reliability of the research conclusion, the standard of data classification was modified, by transferring the physical health from numerical variables to binary variables and if the total score of the six activities was not equal to 6, it will be classified as physically unhealthy, coded as 0. Conversely, if the total score was equal to 6, it will be classified as physically healthy and coded as 1. Furthermore, the measurement of mental health was converted from a binary variable to a continuous variable, and therefore, the total scores of mental health were considered. Furthermore, to supplement the analysis, self-rated health variation was used as a substitute for self-rated health status. The self-rated health variation score ranged from 1 to 5, with a score of 1 indicating very poor health, and a score of 5 indicating very good health. The impact of the health status of older adults on poverty was re-estimated using the panel binary logit regression method. The findings indicate that the initial results remain valid, thus supporting the overall conclusion. More detailed information can be found in Table 4.

**Table 4:** The robustness test.

	*SP*	*OP*
Physical health	−0.251(0.150)	−0.061(0.141)
Mental health	−0.091^***^(0.015)	−0.028^*^(0.014)
Self-rated health	−0.310^***^(0.062)	−0.068(0.059)

Due to space limitations, this article only presents regression coefficients of independent variables. *denotes *p* < 0.05; ***denotes *p* < 0.001.

### 3.5. Analysis of the impact mechanism of health status on poverty

**Figure 1:**
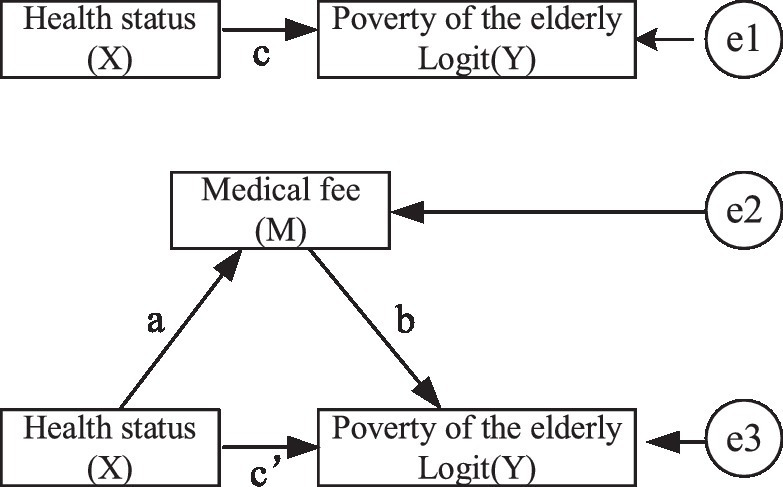
The mediating effect of medical expenses on the relationship between health status and poverty.

Based on the above analysis, it is concluded that the physical, mental, and self-rated health of older adults is associated with both subjective and objective poverty, but the influence mechanism of older adults’ health status on their poverty has not been clarified. It has been suggested that older adults are normally accompanied by multiple morbidities (36, 37), which increases the economic burden of older adults (24, 38). Thus, this ultimately increases the probability of poverty. Therefore, it is assumed that the expenditure of medical expenses is the mediating variable of the influence of health status on subjective and objective poverty in older adults. In this research, the sum of outpatient expenses and hospitalization expenses was taken as the total medical expenses, and then its logarithm was taken as the mediating variable. On the basis of the above discussion, the following research model was constructed (see Figure 1). Logit (Y) represents the probability of poverty of older adults. M is the logarithm of the medical expenses of older adults and X indicates the health status of older adults.


(3) $$Logit\left(Y\right)=cX+e_{1}$$

(4) $$M=aX+e_{2}$$

(5) $$Logit\left(Y\right)=c^{\prime }X+bM+e_{3}$$

This study employed Stata software and stepwise regression analysis to confirm the mediating effect of medical expenses on the relationship between health status and poverty in older adults. Based on the findings from Table 5 for physical health, it can be concluded that the coefficient of c, a, b, and c’ were all statistically significant, indicating that medical expenses act as a mediator in the relationship between physical health and subjective poverty. In other words, poor physical health in older adults may lead to higher medical expenses, which in turn, increases the probability of experiencing subjective poverty. For mental health, the coefficient of regression (a) was not significant, indicating that there was no intermediary effect between medical expenses and subjective poverty. Similarly, for self-rated health, the statistically significant coefficients of variables c, a, b, and c’ suggest that medical expenses played a mediating role in the relationship between self-rated health and subjective poverty. Stated differently, older adults with poor self-rated health are more likely to incur higher medical expenses, which can ultimately contribute to a greater likelihood of experiencing subjective poverty.

**Table 5:** The mediating effect of medical expenses on the relationship between health and poverty.

	Coefficient	*Subjective poverty*	*Objective poverty*
Physical health	c	−0.204^***^(0.053)	NA
	a	−0.120^*^ (0.056)	
	c’	−0.203^***^ (0.053)	
	b	0.046^**^(0.016)	
Mental health	c	−0.670^***^ (0.165)	
	a	0.017 (0.195)	
	c’	−0.675^***^ (0.165)	
	b	0.046^**^ (0.016)	
Self-rated health	c	−0.333^***^(0.059)	−0.127^*^ (0.057)
	a	−0.593^***^ (0.061)	−0.593^***^ (0.061)
	c’	−0.308^***^(0.060)	−0.147^*^ (0.057)
	b	0.046^**^ (0.016)	−0.040^**^(0.014)

*denotes *p* < 0.05; **denotes *p* < 0.01; ***denotes *p* < 0.001.

Likewise, the results indicate that medical expenses served as a mediator in the relationship between self-rated health and objective poverty, as evidenced by the statistically significant coefficients of c, a, b, and c’. In other words, older adults with poor self-rated health are more likely to face higher medical costs, which in turn, can contribute to a decreased probability of experiencing objective poverty.

## 4. Discussion

The primary aim of this study was to empirically examine the impact of health status on poverty among older adults, using three dimensions of health status (physical, mental, and self-rated health) and two dimensions of poverty (subjective and objective). Previous research on the relationship between health status and poverty among older adults has generally been limited to a single dimension.

Based on the analysis results, the following insight can be derived: the health status of older adults, specifically their physical, mental, and self-rated health, has a significant effect on subjective poverty, with higher levels of physical, mental, and self-rated health being associated with lower probabilities of subjective poverty. When older adults are in good physical, mental, and self-rated health, their quality of life and life satisfaction tend to be higher (39). Also, it has been suggested that subjective well-being can be reflected by the so-called subjective poverty concept (40), thus the better one’s health status, the lower the likelihood of experiencing subjective poverty. Furthermore, higher levels of self-rated health were associated with a lower prevalence of objective poverty. One plausible explanation is that self-rated health is a comprehensive indicator of health status, which indicates the true health status, the healthier, the lower frailty, and thus the lower prevalence of absolute poverty (41). However, the analysis did not reveal any significant association between physical health, mental health, and objective poverty. One possible explanation is that when people rate their own health, it gives us a good idea of how healthy they actually are. The better their self-rated health, the less likely they are to suffer from physical frailty, and therefore less likely to experience extreme poverty. This age group of older adults often have savings from their working years (42), or receive pensions or financial support from their children (43), which means that even if they are not healthy, they may not fall into the category of objective poverty. In addition, the most important thing is that due to the existence of social security, older adults have received some economic security (44). Therefore, even if the physical or mental condition of older adults is not good, they will not fall into objective poverty.

We can conclude that there is a correlation between age and both subjective and objective poverty among older adults. As people age, the probability of experiencing subjective poverty tends to decrease, likely due to a decrease in life expectations and an increase in satisfaction with current circumstances (45), which is consistent with a previous study (8). On the other hand, the probability of objective poverty tends to increase with age due to exclusion from the labor market, and thus a decrease in earning capacity, which may increase the odds of living in poverty for older adults (46). Despite experiencing less subjective poverty, older adults remain vulnerable to objective poverty as they age (10).

The residential location has a notable effect on the subjective poverty experienced by older adults. To be more specific, older adults residing in urban areas had a higher likelihood of experiencing subjective poverty than their counterparts in rural areas, which is consistent with a previous study (47). This difference may be attributed to the higher cost of living and increased financial stress in urban areas, while rural areas tend to have lower levels of consumption and less emphasis on material possessions. Additionally, according to an existing study, medication usage is lower among rural individuals compared to those in urban areas (48). As a result, the cost of medication is higher for urban individuals. Consequently, the probability of subjective poverty among older adults living in urban areas is greater.

Furthermore, the marital status of older adults was associated with subjective poverty, with a notably higher incidence of subjective poverty among those with a spouse compared to those without a spouse. This finding contradicts previous research (34). Generally, this may be due to the physical decline that often accompanies aging, which may result in a loss of stable income and increased reliance on adult children for financial support. In societies where being an older adult is viewed as hindering one’s ability to earn money, having an older spouse may be seen as exacerbating poverty. Consequently, the subjective poverty rate among older adults with a spouse is higher than that among those without a spouse.

Furthermore, education was linked to a lower likelihood of subjective poverty among older adults. This may be due to the positive impact of education on self-perception and confidence, leading to a reduced probability of experiencing subjective poverty. In addition, according to the theory of human capital, the early education experience of older adults is the key way to accumulate human capital, which will have a continuous impact on the life of the individual. Previous studies have found that education has a significant positive effect on poverty reduction (49), quality of life (50), and health status (51). On the whole, the higher the education level of older adults, the higher the quality of life and the lower the probability of subjective poverty, which also has been verified in this study.

Smoking and alcohol consumption were found to be unrelated to both subjective poverty and objective poverty among older adults. There was a link between community services and subjective poverty in older adults, possibly due to the provision of services such as personal care, psychological counseling, and home visits. These services, similar to neighborhood services, can give older individuals a feeling of care and support from their community, which can compensate for the inadequacy of individual or household resources and decrease the likelihood of experiencing subjective poverty (18).

In addition, the study found that there was a significant correlation between medical expenses and both subjective and objective poverty among older adults. Specifically, as medical expenses increase, there was a higher probability of experiencing subjective poverty and a lower probability of experiencing objective poverty. High medical expenses can create an economic burden, and subjectively, it may be believed that high medical expenses can lead to poverty (8). However, currently, most people in China have medical insurance, which helps to alleviate the economic pressure of expensive medical expenses. In addition, rapid economic development in China has greatly reduced the incidence of absolute poverty (52), so expensive medical expenses are unlikely to increase the possibility of objective poverty. Moreover, many older people not only participate in basic insurance but may also participate in commercial insurance. Social insurance (a pension) has been found to be a protective factor in alleviating poverty (53, 54). It was found that older persons who have more than one health insurance spent less than those who had coverage only by one health insurance program (44). That is to say, when older adults incur high medical expenses due to illness, they could receive medical compensation from multiple sources, and the amount of compensation received may even exceed the amount paid for medical expenses. This is why medical expenses may sometimes reduce the possibility of objective poverty.

## 5. Contribution and limitations

This article utilizes four waves of longitudinal data to study the impact of the health status of older adults on poverty, and the use of longitudinal data ensures the scientific rigor of the study. Furthermore, this research enriches the relationship research between health and poverty, as previous research mainly focused on the effect of poverty on health. However, this study also has some limitations. Although the latest CLHLS public data were used, the data from 2018 were slightly outdated and did not reflect the most current health and poverty status of older adults. Additionally, this study does not consider debts, which could potentially result in an underestimation of the poverty levels of older adults. Moreover, while the use of mobile communication technology, such as mHealth and eHealth, has been suggested as a means of improving the health status of this population (55), no specific interventions were proposed to address health issues among older adults in this research.

## 6. Implications

Based on the analysis above, the issues of health and poverty among older adults require urgent attention, and in order to effectively alleviate the poverty of older adults, strategies should be taken to improve the health levels of older adults, especially the physical and mental health of high-aged older adults, and the self-rated health of middle-aged older adults. Furthermore, social security and pensions should be further developed to adequately reimburse medical expenditures.

## 7. Conclusion

The primary objective of this study was to empirically analyze the impact of multidimensional health on the multidimensional poverty of older adults using the panel binary logit regression method, with data sourced from the 2008, 2011, 2014, and 2018 CLHLS. Based on the above analysis, the following conclusions can be drawn.

- The findings of this study indicated that the physical, mental, and self-rated health of older adults had a significant positive impact on subjective poverty, while self-rated health also played a critical role in objective poverty.
- There were significant differences in the physical and mental health status among older individuals in different age groups. Specifically, the prevalence of unhealthy physical and mental conditions among older adults was significantly higher in the oldest age group than in both the middle-aged and low-aged groups. Additionally, there are notable disparities in the subjective and objective poverty rates among older individuals in different age groups. In particular, the objective poverty rate in the oldest age group was significantly higher than that observed in the middle-aged and low-aged groups, while the subjective poverty rate in the oldest age group was significantly lower than that observed in the middle-aged and low-aged groups.
- Finally, medical expenditure played a mediation role in the association between health status and poverty of older adults. Specifically, medical expenses mediated the relationship between physical health and subjective poverty, as well as between self-rated health and subjective poverty. Additionally, medical expenses also mediated the relationship between self-rated health and objective poverty.

## Data availability statement

The datasets presented in this study can be found in online repositories. The names of the repository/repositories and accession number(s) can be found in the article/supplementary material.

## Ethics statement

Written informed consent was obtained from the individual(s) for the publication of any potentially identifiable images or data included in this article.

## Author contributions

LZ: data curation. CZ: formal analysis, methodology, and resources. CW: language modification. XZ and LZ: funding acquisition. All authors contributed to the article and approved the submitted version.

## Funding

This study has been supported by the National Natural Science Foundation of China (Grant nos. 71974064 and 71804061), People’s Republic of China.

## Conflict of interest

The authors declare that the research was conducted in the absence of any commercial or financial relationships that could be construed as a potential conflict of interest.

## Publisher’s note

All claims expressed in this article are solely those of the authors and do not necessarily represent those of their affiliated organizations, or those of the publisher, the editors and the reviewers. Any product that may be evaluated in this article, or claim that may be made by its manufacturer, is not guaranteed or endorsed by the publisher.

## Footnotes

^1^United Nations (2012). World Population Prospects: The 2012 Revision. New York, NY: United Nations Department of Economic and Social Affairs. Available online at: http://esa.un.org/unpd/wpp/index.htm.

^2^http://www.xinhuanet.com/politics/2018-07/03/c_1123069056.htm

## References

[1] Wang Z Sun J. Do the poor elderly benefit more than the non-poor elderly from growth? Evidence from urban China. Appl Econ Lett. (2020) 27:836–40. doi: 10.1080/13504851.2019.1646399

[2] Kangas O Palme J. Does social policy matter? Poverty cycles in OECD countries. Int J Health Serv Plan Administr Eval. (2000) 30:335–52. doi: 10.2190/NCWB-35G3-NE2T-8VQR, PMID: 10862379

[3] Gu D Dupre ME Liu G. Characteristics of the institutionalized and community-residing oldest-old in China. Soc Sci Med. (2007) 64:871–83. doi: 10.1016/j.socscimed.2006.10.026, PMID: 17126971

[4] Wang H Kim K Burr JA Wu B. Parent-child relationships and aging Parents' sleep quality: a comparison of one-child and multiple-children families in China. J Aging Health. (2020) 32:1602–13. doi: 10.1177/0898264320947304, PMID: 32772620

[5] Starfield B. Effects of poverty on health status. Bull N Y Acad Med. (1992) 68:17–24. 1555023 PMC1809870

[6] Qu B Li X Liu J Mao J. Analysis of the current situation regarding the aging rural population in China and proposed countermeasures. Popul Health Manag. (2012) 15:181–5. doi: 10.1089/pop.2011.0033, PMID: 22401147

[7] Wang J Liang C Li K. Impact of internet use on elderly health: empirical study based on Chinese general social survey (CGSS) data. Health. (2020) 8:482. doi: 10.3390/healthcare8040482, PMID: 33198418 PMC7712052

[8] Wang H Zhao Q Bai Y Zhang L Yu X. Poverty and subjective poverty in rural China. Soc Indic Res. (2020) 150:219–42. doi: 10.1007/s11205-020-02303-0

[9] Sandoval DA Hirschl R. The increasing risk of poverty across the American life course. Demography. (2009) 46:717–37. doi: 10.1353/dem.0.0082, PMID: 20084826 PMC2831356

[10] Berthoud R Blekesaune M Hancock R. Ageing, income and living standards: evidence from the British household panel survey. Ageing Soc. (2009) 29:1105–22. doi: 10.1017/S0144686X09008605

[11] Morris JL. Explaining the elderly feminization of poverty: an analysis of retirement benefits, health care benefits, and elder care-giving. Notre Dame J Law Ethics Public Policy. (2007) 21:571–608.

[12] Mardiyana LO. The effect of population and education on poverty in East Java 2013-2017. IOP Conf Ser Earth Environ Sci. (2020) 485:012126. doi: 10.1088/1755-1315/485/1/012126

[13] Marchand J Smeeding T. Poverty and aging In: Handbook of the Economics of Population Aging, North-Holland: Elsevier (2016). 905–50.

[14] Collett T Shafer K. Effects of native American geographical location and marital status on poverty. J Sociol Soc Welf. (2016) 43:37–54. doi: 10.15453/0191-5096.3996

[15] Mclanahan SS. Fragile families and the marriage agenda. Frag Fam Marriage Agenda. United States: Springer (2006) 1–21.

[16] Bárcena-Martín E Pérez-Moreno S. Immigrant–native gap in poverty: a cross-national European perspective. Rev Econ Household. (2017) 15:1105–36. doi: 10.1007/s11150-015-9321-x

[17] Qilin Z Yan L. Measurement and decomposition of multidimensional poverty of the elderly in the transitional stage of poverty alleviation——an empirical study based on three periods of CLHLS data. Decis Inform. (2021) 4:61–73. doi: CNKI:SUN:JCYX.0.2021-04-010

[18] Peng C Yip P. Access to Neighbourhood services and subjective poverty in Hong Kong. Appl Res Qual Life. (2022) 18:1015–35. doi: 10.1007/s11482-022-10125-0

[19] Kaida L Boyd M. Poverty variations among the elderly: the roles of income security policies and family co-residence. Can J Aging/La Rev Can Vieilliss. (2011) 30:83–100. doi: 10.1017/S0714980810000814, PMID: 21411026

[20] Sen AK. Development as Freedom. Oxford: Oxford University Press (1999).

[21] Zhu C Walsh C Zhou L Zhang X. Latent classification analysis of leisure activities and their impact on ADL, IADL and cognitive ability of older adults based on CLHLS (2008–2018). Int J Environ Res Public Health. (2023) 20:1546. doi: 10.3390/ijerph20021546, PMID: 36674302 PMC9864528

[22] Angel RJ Frisco M Angel JL Chiriboga DA. Financial strain and health among elderly Mexican-origin individuals. J Health Soc Behav. (2003) 44:536–51. doi: 10.2307/1519798, PMID: 15038148

[23] Maes M. Poverty persistence among the elderly in the transition from work to retirement. J Econ Inequal. (2013) 11:35–56. doi: 10.1007/s10888-011-9200-5

[24] Mueller C Schur C O'Connell J. Prescription drug spending: the impact of age and chronic disease status. Am J Public Health. (1997) 87:1626–9. doi: 10.2105/AJPH.87.10.1626, PMID: 9357343 PMC1381124

[25] Stroupe KT Kinney ED Kniesner TJJ. Does chronic illness affect the adequacy of health insurance coverage? J Health Polit Policy Law. (2000) 25:309–42. doi: 10.1215/03616878-25-2-309, PMID: 10946382

[26] Hua L Zhipeng L. Has the integration of medical insurance for urban and rural residents alleviated the poverty caused by illness in rural areas? Mod Econ Res. (2021) 7:31–9. doi: 10.13891/j.cnki.mer.2021.07.005

[27] Delfani N. Deken J De Dewilde C. Poor because of low pensions or expensive housing? The combined impact of pension and housing systems on poverty among the elderly. Int J Hous Policy (2015) 15: 260–284. doi: 10.1080/14616718.2015.1004880

[28] Grzywacz JG Keyes CLM. Toward health promotion: physical and social behaviors in complete health. Am J Health Behav. (2004) 28:99–111. doi: 10.5993/AJHB.28.2.1 15058511

[29] Lundberg O Manderbacka K. Assessing reliability of a measure of self-rated health. Scand J Soc Med. (1996) 24:218–24. doi: 10.1177/140349489602400314 8878376

[30] Katz SC Ford AB Moskowitz RW Jackson BA Jaffe MW. Studies of illness in the aged. The index of Adl: a standardized measure of biological and psychosocial function. JAMA. (1963) 185:914–9. doi: 10.1001/jama.1963.03060120024016 14044222

[31] World Health Organization Promoting mental health: Concepts, emerging evidence, practice: Summary report. World Health Organization. (2004).

[32] Borg C Hallberg IR Blomqvist K. Life satisfaction among older people (65+) with reduced self-care capacity: the relationship to social, health and financial aspects. J Clin Nurs. (2010) 15:607–18. doi: 10.1111/j.1365-2702.2006.01375.x 16629970

[33] Schneider G Driesch G Kruse A Wachter M Nehen HG Heuft G. What influences self-perception of health in the elderly? The role of objective health condition, subjective well-being and sense of coherence. Arch Gerontol Geriatr. (2004) 39:227–37. doi: 10.1016/j.archger.2004.03.005, PMID: 15381341

[34] Erpeng LIU Qilin Z. Effect evaluation of social endowment insurance on alleviating elderly poverty in rural China——an empirical analysis based on the data CLHLS. J Agrotech Econ. (2011) 2018:98–110. doi: 10.13246/j.cnki.jae.2018.01.009

[35] Dyussenbayev A. Age periods of human life. Adv Soc Sci Res J. (2017) 4:258–263. doi: 10.14738/assrj.46.2924

[36] Bayliss EA Steiner JF Fernald DH Crane LA Main DS. Descriptions of barriers to self-care by persons with comorbid chronic diseases. Ann Fam Med. (2003) 1:15–21. doi: 10.1370/afm.4, PMID: 15043175 PMC1466563

[37] Hall SF. A user's guide to selecting a comorbidity index for clinical research. J Clin Epidemiol. (2006) 59:849–55. doi: 10.1016/j.jclinepi.2005.11.013, PMID: 16828679

[38] Bierman AS Clancy CM. Making capitated Medicare work for women: policy and research challenges. Womens Health Issues. (2000) 10:59–69. doi: 10.1016/S1049-3867(99)00042-0, PMID: 10736559

[39] Wikman A Wardle J Steptoe A. Quality of life and affective well-being in middle-aged and older people with chronic medical illnesses: a cross-sectional population based study. PLoS One. (2011) 6:e18952. doi: 10.1371/journal.pone.0018952, PMID: 21559485 PMC3084723

[40] Nándori S Eszter S. Subjective poverty and its relation to objective poverty concepts in Hungary. Soc Indic Res. (2011) 102:537–56. doi: 10.1007/s11205-010-9743-z

[41] Hayajneh AA Rababa M. The association of frailty with poverty in older adults: a systematic review. Dement Geriatr Cogn Disord. (2021) 50:407–13. doi: 10.1159/000520486, PMID: 34929708

[42] Shefrin HM Thaler RH. The behavioral life-cycle hypothesis. Econ Inq. (1988) 26:609–43. doi: 10.1111/j.1465-7295.1988.tb01520.x

[43] Ha J-H Kahng SK Choi N. Reciprocal effects between health and social support in older adults’ relationships with their children and friends. Res Aging. (2017) 39:300–21. doi: 10.1177/0164027515611182, PMID: 26475653

[44] Kim J Richardson V. The impact of poverty, chronic illnesses, and health insurance status on out-of-pocket health care expenditures in later life. Soc Work Health Care. (2014) 53:932–49. doi: 10.1080/00981389.2014.955940, PMID: 25397347

[45] Chen Y Peng Y Fang P. Emotional intelligence mediates the relationship between age and subjective well-being. Int J Aging Hum Dev. (2016) 83:91–107. doi: 10.1177/0091415016648705, PMID: 27199490 PMC5442987

[46] Lee Y Hong PY Harm Y. Poverty among Korean immigrant older adults: examining the effects of social exclusion. J Soc Serv Res. (2014) 40:385–401. doi: 10.1080/01488376.2014.894355

[47] Lee GR Lassey ML. Rural-urban differences among the elderly: economic, social, and subjective factors. J Soc Issues. (1980) 36:62–74. doi: 10.1111/j.1540-4560.1980.tb02022.x

[48] Tam HL Chung SF Wang Q. Urban-rural disparities in hypertension management among middle-aged and older patients: results of a 2018 Chinese national study. Chronic Illness. (2022) 0:174239532211026. doi: 10.1177/17423953221102627 35603631

[49] Awan MS Malik N Sarwar H Waqas M. Impact of education on poverty reduction. Int J Acad Res. (2011) 3:659–64.

[50] Schwartz RM Bevilacqua KG Alpert N Liu B Dharmarajan KV Ornstein KA. Educational attainment and quality of life among older adults before a lung Cancer diagnosis. J Palliat Med. (2020) 23:498–505. doi: 10.1089/jpm.2019.0283, PMID: 31702439 PMC7718850

[51] Xiang L Xindong Z. How does education affect the health level of the elderly in China? J Financ Econ. (2020) 46:139–53. doi: 10.16538/j.cnki.jfe.2020.03.010

[52] Zhou Y Guo Y Liu Y Wu W Li Y. Targeted poverty alleviation and land policy innovation: some practice and policy implications from China. Land Use Policy. (2018) 74:53–65. doi: 10.1016/j.landusepol.2017.04.037

[53] Peeters H Tavernier D Wouter A. Lifecourses, pensions and poverty among elderly women in Belgium: interactions between family history, work history and pension regulations. Ageing Soc. (2015) 35:1171–99. doi: 10.1017/S0144686X14000129

[54] Choi YJ Kim JW. Contrasting approaches to old-age income protection in Korea and Taiwan. Ageing Soc. (2010) 30:1135–52. doi: 10.1017/S0144686X10000413

[55] Wong EM Leung DYP Tam HL Wang Q Yeung KW Leung AYM. Effectiveness of a nurse-led support Programme using a Mobile application versus phone advice on patients at risk of coronary heart disease–a pilot randomized controlled trial. Risk Manag Healthcare Policy. (2022) 15:597–610. doi: 10.2147/RMHP.S355554, PMID: 35422666 PMC9005123

